# Dendrite Morphology Minimally Influences the Synaptic Distribution of Excitation and Inhibition in Retinal Direction-Selective Ganglion Cells

**DOI:** 10.1523/ENEURO.0261-21.2021

**Published:** 2021-09-07

**Authors:** Malak El-Quessny, Marla B. Feller

**Affiliations:** 1 Helen Wills Neuroscience Institute, University of California, Berkeley, Berkeley, CA, 94720; 2 Department of Molecular and Cell Biology, University of California, Berkeley, Berkeley, CA, 94720

**Keywords:** dendrites, direction selective, morphology, receptive field, retina, synaptic inputs

## Abstract

Throughout the nervous system, the organization of excitatory and inhibitory synaptic inputs within a neuron’s receptive field shapes its output computation. In some cases, multiple motifs of synaptic organization can contribute to a single computation. Here, we compare two of these mechanisms performed by two morphologically distinct retinal direction-selective ganglion cells (DSGCs): directionally tuned inhibition and spatially offset inhibition. Using drifting stimuli, we found that DSGCs that have asymmetric dendrites exhibited stronger directionally tuned inhibition than symmetric DSGCs. Using stationary stimuli to map receptive fields, we found that DSGCs with both symmetric and asymmetric dendrites exhibited similar spatially offset inhibition. Interestingly, we observed that excitatory and inhibitory synapses for both cell types were locally correlated in strength. This result indicates that in the mouse retina, dendritic morphology influences the amount of tuned inhibition attained through asymmetric wiring but does not dictate the synaptic organization of excitation relative to inhibition.

## Significance Statement

Neural circuit function is dependent on the detailed organization of excitatory and inhibitory synapses onto dendrites. Here, we use a classic neural circuit, the direction-selective circuit of the retina, to assess how changes in dendritic shape impact the synaptic organization. We find the direction-selective cells of the retina that have asymmetric dendrites have similar synaptic organization to those that have symmetric dendrites, indicating the shape of dendrites does not dictate the final computation of the neurons.

## Introduction

Detecting the direction of moving stimuli is an essential part of sensory processing. In the mouse visual system, direction selectivity is first observed in the retina, where direction-selective ganglion cells (DSGCs) fire many action potentials in response to motion in their preferred direction, and few to no action potentials in response to the opposite, or null, direction. Direction-selective computations occur across many layers of the mammalian visual system from DSGCs of the retina, to the retino-recipient neurons of the dorsal lateral geniculate nucleus (dLGN) of the thalamus (Marshel et al., 2012; Liang et al., 2018), thalamo-recipient layer four neurons and intracortical circuits of the visual cortex (Rasmussen et al., 2020; Rossi et al., 2020). Additionally, direction selectivity has been shown to arise in nonvisual areas like the mouse whisker somatosensory cortex (Laboy-Juárez et al., 2019) and in the primary auditory cortex (Zhang et al., 2003; Ye et al., 2010).

Retinal direction selectivity is mediated primarily by inhibition through two non-mutually exclusive mechanisms. The first mechanism is based on directional tuning of inhibition, where the amount of inhibitory input onto a DSGC is greater for null direction motion than for preferred direction motion. In the mammalian retina, this tuned inhibition is provided by starburst amacrine cells (SACs), where the combination of SAC centrifugal directional tuning (Gavrikov et al., 2006; Hausselt et al., 2007; Ding et al., 2016; Vlasits et al., 2016), and DSGC-SAC asymmetric wiring (Briggman et al., 2011; Wei et al., 2011; Yonehara et al., 2011; Rosa et al., 2016), ensures maximal spike suppression in response to null direction motion, compared with preferred direction motion. Although the role of tuned inhibition in generating direction-selective responses has been well established in the mouse and rabbit (Fried et al., 2002; Taylor and Vaney, 2002; Wei et al., 2011; Yonehara et al., 2011; Grama and Engert, 2012; Morrie and Feller, 2015), its dependence on the morphology of DSGCs has been relatively unexplored.

The second mechanism is based on spatially offset inhibition, a term used to describe when excitatory and inhibitory receptive fields are spatially offset from each other. Hence, during preferred direction motion, the stimulus elicits an excitatory response before an inhibitory response, allowing the cell the fire action potentials. During null direction motion, the stimulus elicits an inhibitory response primarily at the same time as the inhibitory response, effectively suppressing spiking output. This is the classic mechanism postulated to underlie direction-selective responses in both the retina (Fried et al., 2002; Yonehara et al., 2011) and in the visual cortex (Hubel and Wiesel, 1959, 1962; Priebe and Ferster, 2005; Li et al. 2015; Wilson et al. 2018; Rossi et al., 2020). Several studies have revealed that temporal delays play a role in the DS computation of the mouse retina, consistent with the presence of spatially offset inhibition (Hanson et al., 2019; Pei et al., 2015; Ding et al., 2021). Recently, we used receptive field mapping to show that a population of asymmetric, ventral preferring DSGCs (vDSGCs) have both tuned inhibitory inputs and spatially offset inhibition, although neither of these circuit contributions were impacted by dramatic changes in the dendrite orientation because of dark-rearing (El-Quessny et al., 2020). However, how spatially offset inhibition depends on dendritic morphology is not known.

Anatomical studies indicate that ON-OFF DSGCs exhibit a uniform distribution of GABA_A_ receptors on their dendrites (Auferkorte et al., 2012; Sigal et al., 2015; Bleckert et al., 2018), while functional studies indicate that SACs whose somas are located on the null side of a DSGC provide stronger inhibitory drive than SACs located on the preferred side of DSGC asymmetric wiring (Lee et al., 2010; Wei et al., 2011; Morrie and Feller, 2015). Here, we compare the organization of excitatory and inhibitory receptive fields of two subsets of DSGCs that have distinct morphologies. The first is a subset of vDSGCs, which have asymmetric dendrites that are oriented toward their preferred direction (Trenholm et al., 2011), a configuration which contributes to their direction selectivity in the absence of inhibitory input (Trenholm et al., 2011; El-Quessny et al., 2020). The second is a subset of nasal motion preferring DSGCs (nDSGCs), which have symmetric dendrites that are not oriented in any particular direction (Rivlin-Etzion et al., 2011). Multielectrode array data has shown that the spiking output of both DSGC subtypes possesses similar directional tuning under bright stimulus conditions (Yao et al., 2018). Here, we combine morphologic reconstructions with whole cell voltage clamp recordings to show that asymmetric vDSGCs have sharper tuning of inhibition relative to symmetric nDSGCs. Additionally, we map the receptive fields of both DSGC subtypes, using stationary stimuli, and show no difference in the spatial offset of inhibition relative to excitation despite distinct dendritic morphologies.

## Materials and Methods

### Experimental model and subject details

Mice used in this study were aged from postnatal day 30 (p30) to p60 and were of both sexes. Animals used in experiments had not previously been involved in other experiments or exposed to any drugs. Animal health was monitored daily and only healthy animals were used in experiments. To target vDSGCs, we used Hb9::GFP (Arber et al., 1999) mice, which express GFP in a subset of vDSGCs which have asymmetric dendrites (Trenholm et al., 2011). To target nDSGCs, we used Trhr::GFP mice (Rivlin-Etzion et al., 2011). All experiments involved recording from one to seven cells from at least two animals of either sex. All animal procedures were approved by the University of California, Berkeley Institutional Animal Care and Use Committee and conformed to the National Institutes of Health *Guide for the Care and Use of Laboratory Animals*, the Public Health Service Policy, and the Society for Neuroscience Policy on the Use of Animals in Neuroscience Research.

### Method details

#### Retina preparation

Mice were anesthetized with isoflurane and decapitated. Retinas were dissected from enucleated eyes in oxygenated (95% O_2_/5% CO_2_) Ames’ media (Sigma) for light responses. Retinal orientation was determined as described previously (Wei et al., 2010). Isolated whole retinas were micro-cut at the dorsal and ventral halves to allow flattening, with dorsal and ventral mounted over a 1- to 2-mm^2^ hole in nitrocellulose filter paper (Millipore) with the photoreceptor layer side down and stored in oxygenated Ames’ media until use (maximum 10 h). All experiments were performed on retinas in which dorsal-ventral orientation was tracked.

#### Visual stimulation

For visual stimulation of DSGCs, visible light (420–530 nm) were generated using a computer running 420- to 520-nm light through a digital micro-mirror device (DLI Cel5500) projector with a light emitting diode (LED) light source generated using MATLAB software with the Psychophysics Toolbox. Visual stimuli are focused on the photoreceptor layer using a condenser in the DMD path to the chamber.

##### Moving stimuli

To measure the directional tuning of synaptic currents onto DSGCs, drifting bars of positive contrast on a gray background (96% Michaelson’s contrast) were presented (velocity = 250 μm/s, length = 600 μm, width = 350 μm over a 700-μm radius circular mak) in eight block shuffled directions, repeated three times, moving along the long axis of the bar. Each presentation lasted 6 s and was followed by 3 s interstimulus interval of gray background. For these moving stimuli, the illumination radius on the retina was 1.4 mm to limit modulation of DSGC responses by inhibitory wide-field amacrine cells (Chen et al., 2016). A 20× water-immersion objective (Olympus LUMPlanFl/IR 360/1.0 NA) was used to target cells for voltage clamp recordings, which were simultaneously acquired using methods described below.

##### Static stimuli for receptive field mapping

To map excitatory and inhibitory receptive fields of DSGCs, positive contrast square stimuli (30 × 30 μm) were flashed over a gray background (96% Michaelson’s contrast) at an intensity of 3.1 × 10^5^ R*/s/rod. Stimuli were individually presented in 100 block-shuffled positions, repeated three times, with each stimulus lasting for 0.5 s followed by a 1.2 s interstimulus interval of gray background. Stimuli were presented within a 10 × 10 grid, onto a stimulus field of 500 × 500 μm, with the DSGC soma located in the center of the stimulus field.

**Figure 1.**
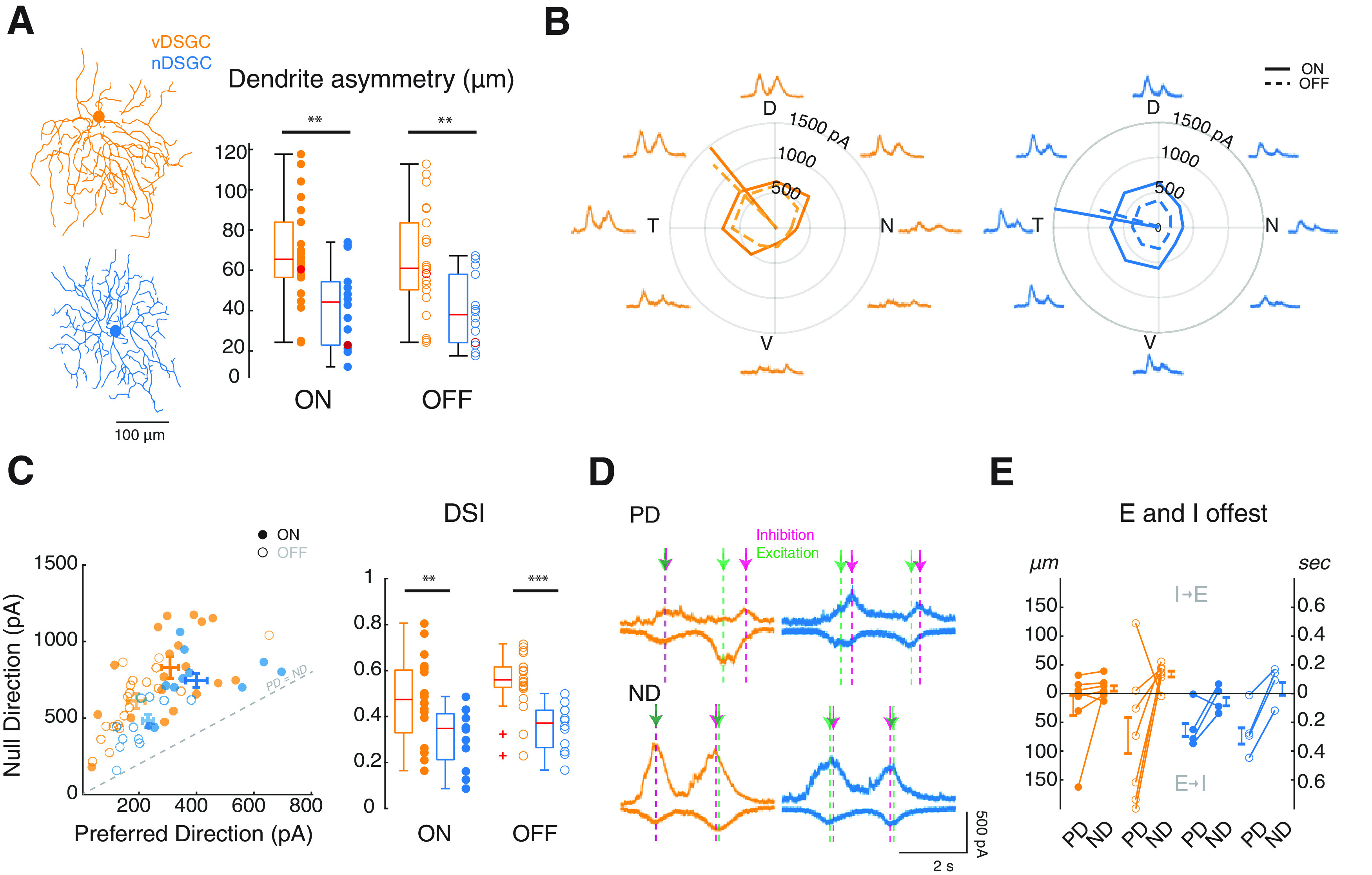
DSGCs with asymmetric dendrites exhibit more asymmetric inhibition but similar temporal offset compared to DSGCs with symmetric dendrites. ***A***, Left, Skeletonized vDSGC (orange) and nDSGC (blue). Right, Summary data comparing dendritic asymmetry of vDSGCs (*n* = 23) and nDSGCs (*n* = 16) as measured by the magnitude of the vector from the soma to COM of the ON (filled) and OFF (open) dendrites. Red data points indicate the measurements for example cells on the left. Statistical significance assessed by one-way ANOVA (*p* = 4 × 10^–4^) and Dunn–Sidak *post hoc* test (**p < 0.01). ***B***, Example tuning curve and mean traces of the IPSCs in vDSGCs (orange, left) and nDSGCs (blue, right) in response to a moving bar stimulus. ON (solid lines) and OFF (dashed lines) tuning curves and vector sums are based on peak current amplitudes. ***C***, Left, Scatter plot of the peak amplitude of IPSCs in response to preferred versus null direction motion in vDSGCs (orange) and nDSGCs (blue). SEM for ON (dark shade) and OFF (light shade) responses indicated on the plot. Right, Summary data presenting the tuning of vDSGCs (orange) and nDSGCs (blue) as measured by the direction selectivity index. ON (filled) and OFF (open) responses separately. Unity line (gray dashed) indicating where preferred (PD) = null (ND) IPSC peak amplitude. Statistical significance assessed by Wilcoxon rank-sum test (**p < 0.01, ***p < 0.001). ***D***, Example IPSC and EPSC traces in response to the preferred direction (PD, top) and null direction (ND, bottom) for a vDSGC (orange) and a nDSGCs (blue). Arrows indicating peak timing for IPSCs (magenta) and EPSCs (green). ***E***, Summary data representing spatial offset based on the timing differences of the peak excitatory (E) and inhibitory (I) currents in response to preferred direction (PD) and null direction (ND) stimulation for ON (filled) and OFF (open) responses in vDSGCs (orange) and nDSGCs (blue). Statistical significance for nDSGCs assessed by paired *t* test (*p* > 0.05).

#### Two-photon targeted whole-cell voltage-clamp recordings

Oriented retinas were placed under the microscope in oxygenated Ames’ medium at 32–34°C. Identification and recordings from GFP+ cells were performed as described previously (Wei et al., 2010). In brief, GFP+ cells were identified using a custom-modified two-photon microscope (Fluoview 300; Olympus America) tuned to 920 nm to minimize bleaching of photoreceptors. The inner limiting membrane above the targeted cell was dissected using a glass electrode. Cell attached voltage clamp recordings were performed with a new glass electrode (4–5 MΩ) filled with internal solution containing the following: 110 mm CsMeSO_4_, 2.8 mm NaCl, 20 mm HEPES, 4 mm EGTA, 5 mm TEA-Cl, 4 mm Mg-ATP, 0.3 mm Na_3_GTP, 10 mm Na_2_phosphocreatine, and 5 mm QX-Cl (pH 7.2 with CsOH, osmolarity = 290, ECl^–^ = −60 mV). Whole cell recordings were performed with the same pipette after obtaining a giga ohm (1 GΩ) seal and breaking into the cell membrane. Holding voltages for measuring excitation and inhibition after correction for the liquid junction potential (10 mV) were 0 and −70 mV, respectively. Signals were acquired using Clampex 10.4 recording software and a Multiclamp 700A amplifier (Molecular Devices), sampled at 10 kHz, and low pass filtered at 6 kHz.

#### Two-photon imaging and morphologic reconstruction

After physiological recordings of DSGCs were completed, Alexa Fluor 594-filled DSGCs were imaged using two-photon excitation at 800 nm. At this wavelength, GFP is not efficiently excited, but Alexa Fluor 594 is brightly fluorescent; 480 × 480 μm Image stacks were acquired at z intervals of 1.0 μm and resampled fifteen times for each stack using a 20× objective (Olympus LUMPlanFl/IR 2× digital zoom, 1.0 NA) 30 kHz resonance scanning mirrors covering the entire dendritic fields of the DSGCs. Image stacks of DSGCs were then imported to FIJI (NIH) and a custom macro was used to segment ON and OFF dendrites based on their lamination depth in the inner plexiform layer (ON layer 10–30 μm, OFF layer 35–55 μm in depth). Following ON and OFF dendritic segmentation, we used the Simple Neurite Tracer plugin in FIJI to skeletonize and then binarize the ON and OFF dendritic segments for morphologic analyses.

#### Pharmacology

For experiments conducted in Hexamethonium (Millipore Sigma), we diluted 100 μm in Ames’ media, and allowed it to perfuse for 5–10 min at a perfusion rate of 1 ml/min.

### Quantification and statistical analysis

#### Statistical tests

Mean ± SDs for all angles performed using circular mean and circular SDs. Details of statistical tests, number of replicates, and *p* values are indicated in the figures and figure captions; *p* < 0.05 was considered significant.

#### Data analysis

For voltage clamp recordings during moving stimuli, traces were first averaged across the three trials for each direction and inspected to ensure the consistency of the responses across trials. Average traces were baseline subtracted based on the last 500 ms of recording or a user defined interval after manual inspection. Peak currents were calculated from average baseline subtracted traces. They were defined as the maximal (IPSC) or minimal (EPSC) points during two separate 1.9 s windows in which the ON and OFF responses occurred. The peak currents in each direction were used to calculate the vector sum of the current responses. For timing analysis, PSC traces were low pass filtered using an 80-ms moving average, and the peak times for excitation and inhibition was extracted for both ON and OFF responses. Null directions for both ON and OFF responses were defined as the angle of the vector sum of ON and OFF peak IPSCs; the preferred directions were defined as 180° from the, null direction.

The directionally selective index (DSI) was calculated for the peak amplitude of the IPSCs as: (ND – PD)/(ND + PD), where ND is the amplitude of the peak current in the null direction, and PD is the amplitude of the peak current in the preferred direction. We also used the magnitude of the vector sum of the PSCs as another measurement of directional tuning (vector sum = 1 – circular variance of the PSCs; Mazurek et al., 2014).

##### Quantification of receptive fields

For voltage clamp recordings during static stimuli, we first divided each trace into the ON and OFF response based on the location of the stimulus. Next, we calculated the center of mass (COM) of the receptive field using the following equations:

$${\mathrm{COM}}_{x}=\frac{\sum_{i=1}^{N}m_{i}x_{i}}{M}$$

$${\mathrm{COM}}_{y}=\frac{\sum_{i=1}^{N}m_{i}y_{i}}{M}$$

Where *x* and *y* are the cartesian coordinates of the COM, *N* is the total number of stimulus squares (100), *m* is the peak current amplitude at each coordinate location, and *M* is the sum of peak current amplitudes across the entire receptive field.

To measure the displacement and orientation of the receptive fields relative to the soma, we calculated the magnitude and angle, respectively, of vector from the soma to the COM of the receptive field using the following equations:

Vector magnitude = $\surd {\vec{A}}_{x}{}^{2}+{\vec{A}}_{y}{}^{2}$, where ${\vec{A}}_{x}$ and ${\vec{A}}_{y}$ are the vector components from the soma to COM_x_ and COM_y_, respectively.

$$\text{Vector Angle }={\text{tan}}^{-\text{1}}\left(\frac{{\vec{A}}_{x}}{{\vec{A}}_{y}}\right).$$

To quantify spatially offset inhibition, we calculated the vector from the COM of the excitatory receptive field to the COM of the inhibitory receptive field.

##### Quantification of dendrite asymmetry

**Figure 2.**
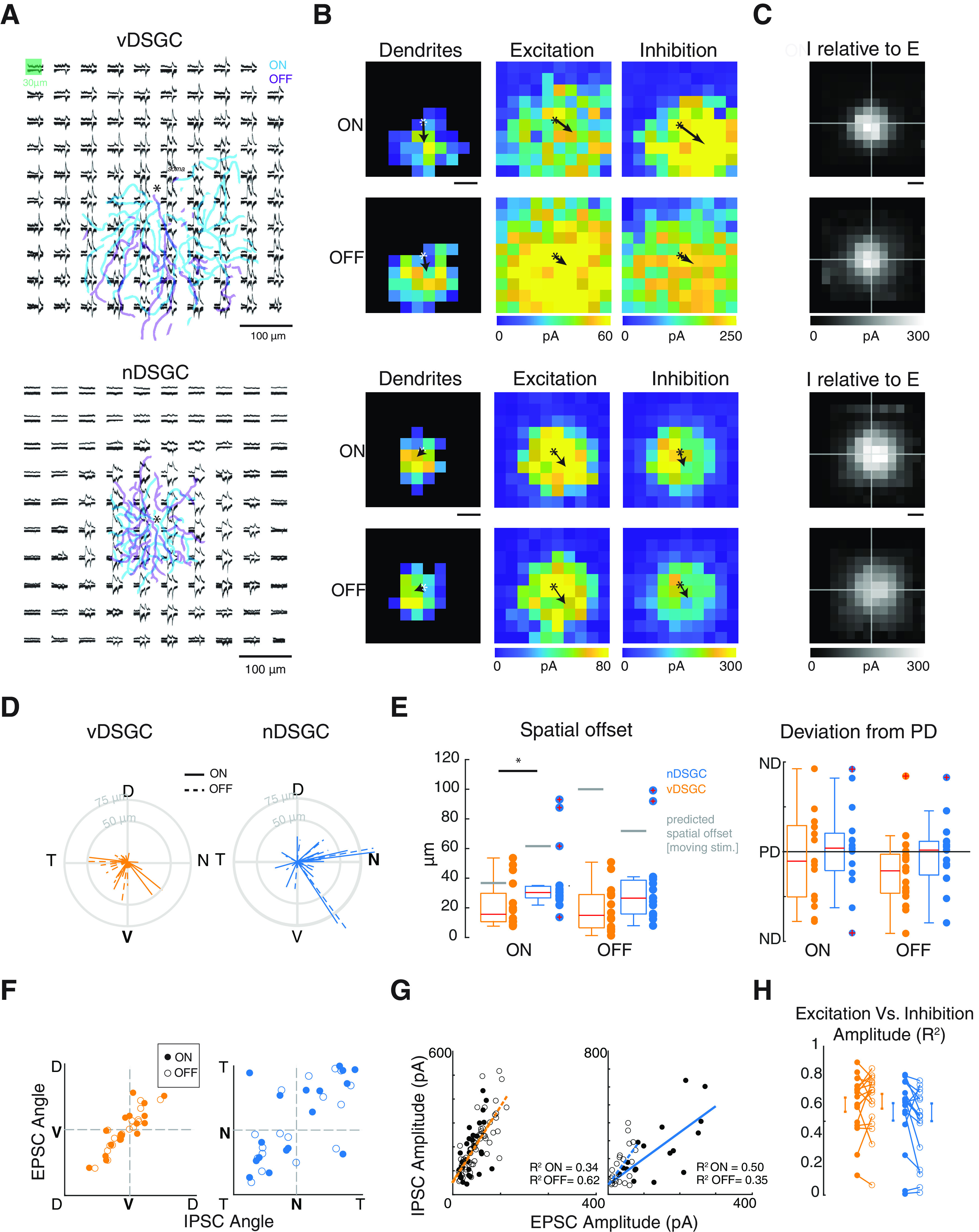
vDSGCs and nDSGCs have similar spatially offset inhibition and exhibit strong local correlations in excitation and inhibition. ***A***, Example vDSGC (top) and nDSGCs (bottom) receptive field displaying mean inhibitory and excitatory postsynaptic responses for each stimulus presentation. Asterisk in the center of the stimulus field denotes soma location. ON (cyan) and OFF (purple) dendritic skeletons are overlaid. Inset shows stimulus presentation of 30 × 30 μm light within a 50 × 50 μm area to evade scattering effects. ***B***, Heat map of dendritic density (left), the EPSC peak current amplitude (middle), and IPSC peak current amplitude (right) for ON (top) and OFF (bottom) responses of the example vDSGC (top row) and nDSGC (bottom row) to the left. Scale bar: 100 μm. ***C***, Summary data plotting the average IPSC (I) receptive field centered on the EPSC (E) receptive field COM (ECOM). ON (top) and OFF (bottom) responses are analyzed separately. Scale bar: 100 μm. ***D***, Summary data represented as polar plots of the vectors from the excitatory (center) to the inhibitory receptive fields in vDSGCs (orange, left) and nDSGCs (blue, right) for ON (solid) and OFF (dashed) responses. ***E***, Left, Summary data showing magnitude of spatially offset inhibition (vector from E to I) for vDSGCs (orange) and nDSGCs (blue). Spatial offset predicted from the temporal offset of excitation and inhibition during preferred direction motion of a moving bar stimulus (Fig. 1) indicated in gray. Statistical significance across cell types assessed with Wilcoxon rank-sum test (*p < 0.05). Statistical significance between measured and predicted spatial offset determined by one-sided *t* test (all *p* values <0.001). Right, Summary data showing the angular deviation of spatially offset inhibition from the preferred direction of vDSGCs (orange) and nDSGCs (blue). ***F***, Summary data representing the orientation of the EPSC’s receptive field COM relative to the orientation of the IPSC’s receptive field COM in vDSGCs (orange, top) and nDSGCs (blue, bottom) for ON (filled) and OFF (open) responses. Pearson’s correlation coefficients presented in Table 2. ***G***, Example scatter plots of EPSC versus IPSC amplitude per pixel in vDSGCs (orange, left) and nDSGCs (blue, right) for ON (filled) and OFF (open) responses. Trend lines computed using least squares regression. Pixels with current amplitude below 5% of the maximum were excluded. Inset, Coefficient of determination (*R*^2^) for each example cell. ***H***, Summary data of *R*^2^ values for each vDSGC (orange) and nDSGC (blue).

To compare DSGC dendrites to the synaptic inputs evoked by static stimuli, we skeletonized dendrites using methods described above. Next, we calculated the vector from soma to the COM of the dendritic pixels; the magnitude of the vector indicates the magnitude of dendritic asymmetry relative to the soma, while the angle of the vector indicates the orientation dendrites. To directly compare the DSGC dendrites to the IPSC and EPSC receptive fields, we binned the dendritic skeleton into a 10 × 10 matrix by summing the binarized pixels in each bin, with the soma located in the center of the matrix. Again, we calculated the vector from soma to the COM of the binned dendritic pixels; the magnitude of the vector indicates the magnitude of dendritic asymmetry relative to the soma, while the angle of the vector indicates the orientation dendrites. In Figure 2*A*, we show the responses of an example vDSGC and nDSGC recorded in control conditions as well as their binned dendrites.

##### Quantification of receptive and dendritic field sizes

To quantify receptive field size, the locations in the excitatory and inhibitory pixels that had responses below a set noise threshold of 50 pA were assigned a value of 1 while the other pixels were assigned a value of zero. Next, we calculated the area total area of the grid with responses that exceed the threshold. To quantify dendritic field size in a manner that is comparable to the receptive field size, we binned dendritic pixels into a 10 × 10 matrix and then used the same method to calculate dendritic area, without applying a threshold since dendrites were skeletonized before this analysis using the method described above.

## Results

### DSGCs with asymmetric dendrites exhibit greater directional tuning of inhibition than DSGCs with symmetric dendrites

Our goal was to determine if the synaptic organization onto DSGCs is dependent on the dendritic morphology. We first quantified the difference in dendritic asymmetry in vDSGCs versus nDSGCs by calculating the magnitude of the vector from the soma to the COM of the dendritic pixels. We found that both ON and OFF dendrites of vDSGCs were significantly more asymmetric than nDSGCs (Fig. 1*A*, Table 1). As reported previously, the asymmetry in the dendrites of nDSGCs are not consistently aligned with their preferred direction (Rivlin-Etzion et al., 2011).

To assess the impact of dendrite morphology on the tuning of inhibition, we conducted voltage clamp recordings of both vDSGCs and nDSGCs and isolated IPSCs in response to a bar of light moving in eight different directions (Fig. 1*B*). Despite previous MEA studies showing that comparable spike tuning of both DSGC subtypes under our stimulus conditions (Yao et al., 2018), asymmetric vDSGCs had a significantly higher DSI, compared with nDSGCs (Fig. 1*C*, Table 1). Hence, vDSGCs with asymmetric dendrites had greater tuning of inhibition.

Previous studies have reported differences in the relative timing of excitatory and inhibitory synaptic inputs for preferred and null direction stimulation, consistent with the presence of spatially offset inhibition (Fried et al., 2002; Taylor and Vaney, 2002). Here, we report similar differences in timing, with inhibitory inputs delayed relative to excitatory input for preferred direction stimulation in symmetric, nDSGCs (Figure 1*D*, Table 1), although there was greater variability during preferred direction motion for asymmetric vDSGCs because of the small amplitude of the inhibitory currents (Fig. 1*E*). For both nDSGCs and vDSGCs, null direction motion elicited a much smaller temporal difference between the excitatory and inhibitory responses (Figure 1*D*, Table 1). We also represented these timing differences as spatial offsets by multiplying by the velocity of our stimulus (250 μm/s = 8.1°/s) (Figure 1*E*, Table 1). These data suggest that, for both asymmetric vDSGCs and symmetric nDSGCs, spatially offset inhibition contributes to the DS computation.

**Table 1:** Summary data for Figure 1

	ON responses				OFF responses			
	vDSGCs		nDSGCs		vDSGCs		nDSGCs	
	Mean	SD (n)	Mean	SD (n)	Mean	SD	Mean	SD
Dendrite asymmetry (μm)	66.67	25.50 (23)	43.08	20.51 (17)	65.83	25.40	39.98	17.32
Dendrite angle (°)	242.11	41.70 (23)	146.87	67.12 (14)	230.80	40.56	234.14	76.06
IPSC amplitude (ND; pA)	794.60	309.80 (17)	665.99	146.30 (11)	574.92	181.10	449.37	102.50
IPSC amplitude (PD; pA)	290.70	136.81 (17)	325.75	101.98 (11)	173.58	88.61	212.96	63.23
IPSC DSI	0.48	0.19 (17)	0.34	0.14 (11)	0.56	0.11	0.31	0.17
E–I timing difference (ND; ms)	36.04	67.30 (7)	−53.11	92.43 (5)	136.22	76.41	39.66	144.91
E–I timing difference (PD; ms)	81.63	−262.06 (7)	−257.57	134.26 (5)	−292.43	476.53	−173.50	325.06
E–I spatial offset (ND; μm)	13.32	13.07 (7)	21.77	12.73 (5)	35.31	16.23	33.52	5.58
E–I spatial offset (PD; μm)	36.04	57.12 (7)	64.39	33.57 (5)	109.55	76.79	74.67	44.58

Table related to Figure 1. ND, Null Direction; PD, Preferred Direction.

### Receptive field mapping of DSGCs reveals similar spatially offset inhibition for DSGCs with symmetric or asymmetric dendrites

Previously, we showed that in asymmetric vDSGCs, the centers of mass of the spatial receptive fields for excitation and inhibition are both offset toward the preferred direction with inhibitory receptive fields further offset than the excitatory receptive fields (El-Quessny et al., 2020). However, for symmetric nDSGCs, the relative arrangement of excitatory and inhibitory receptive fields is unknown. Hence, we mapped the excitatory and inhibitory receptive fields by recording synaptic currents evoked by squares of light sequentially presented at 100 block-shuffled locations within a soma-centered grid (Fig. 2*A*). We stimulated a 500 × 500 μm area spanned by a 10 × 10 grid and we presented a 30 × 30 μm light flash within the center of each grid to prevent any blooming artifacts of the visual stimulus.

To characterize the relative position of excitatory and inhibitory receptive fields, we computed the COM for dendrites, excitatory receptive fields, and inhibitory receptive fields (Fig. 2*B*) and compared both the relative displacement and orientation of the inhibitory receptive field to the excitatory receptive field (Fig. 2*C*). We found that the excitatory and inhibitory receptive fields of both vDSGCs and nDSGCs exhibited some spatial offset (Fig. 2*D*). Although the relative magnitude of spatially offset inhibition (magnitude of the vector from excitation to inhibition) was slightly greater in nDSGCs, compared with vDSGCs (Table 2,  Fig. 2*E*), we were surprised to find that they were comparable to each other despite their distinct dendritic morphologies. Moreover, we observed that the direction of the spatially offset inhibition clustered around the preferred direction though there was significant variance for both nDSGCs, and vDSGCs (Table 2; Fig. 2*D*,*E*).

**Table 2:** Summary data for Figure 2

	ON Responses				OFF Responses			
	vDSGCs (n=17)		nDSGCs (n=15)		vDSGCs		nDSGCs	
	Mean	SD	Mean	SD	Mean	SD	Mean	SD
RF spatial offset magnitude (E–I; μm)	20.80	15.54	38.14	23.39	19.83	14.49	33.72	27.01
RF spatial offset deviation from PD (°)	−7.53	86.59	7.90	86.95	−40.79	72.278	−4.97	70.20
	*R*	*p*	*R*	*p*	*R*	*p*	*R*	*p*
EPSC angle vs IPSC angle	0.83	2.7E-05	0.67	5.0E-03	0.92	1.6E-09	0.67	6.3E-03

Table related to Figure 2.

*R*: Pearson’s correlation.

*p*: *p* value.

Although we observed a shift in the position of inhibitory receptive fields relative to excitatory receptive fields in both cell types, there was also a striking correlation between them. First, we observed a strong positive correlation between the location of excitation and inhibition relative to the soma (Fig. 2*F*). Note, this correlation was stronger in asymmetric vDSGCs (Table 2) consistent with previous findings (El-Quessny et al., 2020). Second, we observed a strong correlation between the strength of excitation and inhibition measured at each pixel (Fig. 2*G*), where the amplitude of excitation explains on average 65% and 51% of the variance in the amplitude of inhibition in vDSGCs and nDSGCs, respectively (Fig. 2*H*). This strong local correlation is consistent with the tight alignment of ACh-GABA co-transmission from SAC varicosities (Lee et al., 2010; Sethuramanujam et al., 2016; Brombas et al., 2017; Jain et al., 2020).

**Figure 3.**
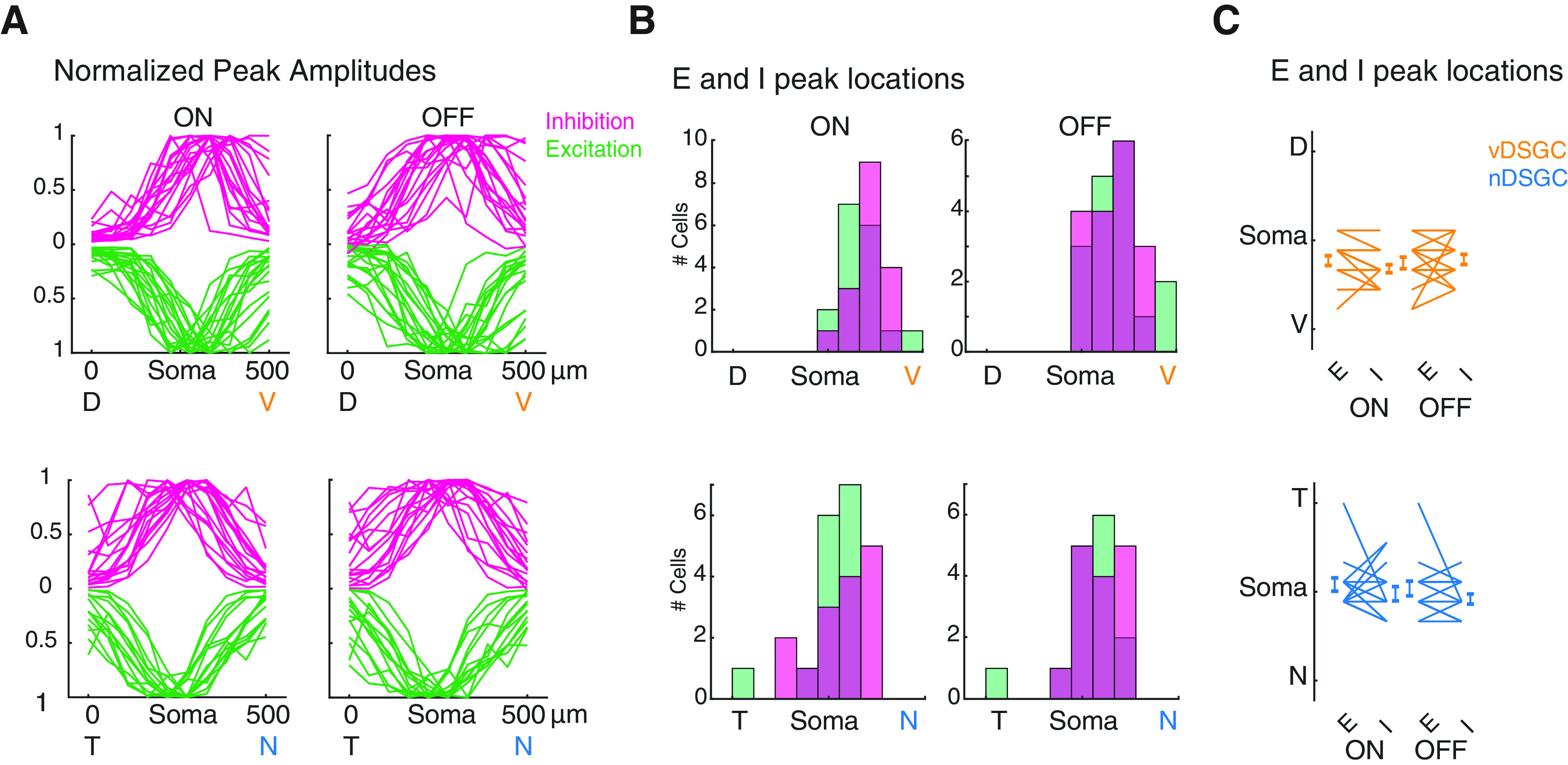
vDSGCs and nDSGCs display comparable distribution of synaptic inputs along their preferred-null axis. ***A***, Summary data displaying the normalized amplitude of the inhibitory (magenta) and excitatory (green) inputs along the null-preferred axis of vDSGCs (top, *n* = 17 cells) and nDSGCs (bottom, *n* = 15 cells). ON (left) and OFF (right) responses plotted separately. ***B***, Summary data representing the distribution of the locations of the peak inhibitory (magenta) and excitatory (green) inputs along the null-preferred axis of vDSGCs (top) and nDSGCs (bottom). ***C***, Summary data representing the locations of the peak excitatory (E) and inhibitory (I) inputs along the null-preferred axis of vDSGCs (orange, top) and nDSGCs (blue, bottom). Statistical significance determined with a paired *t* test (*p* > 0.05).

To assess the organization of the excitatory and inhibitory receptive fields along the preferred-null axis, we collapsed the synaptic currents recorded with the static stimulus along the axis orthogonal to their preferred direction and plotted the normalized distribution of excitation and inhibition (Fig. 3*A*). We found both vDSGCs and nDSGCs exhibit a comparable skew in the spatial distribution of excitatory and inhibitory synapses toward their preferred directions (Fig. 3*B*,*C*). Together, these data indicate that nDSGCs and vDSGCs exhibited similar spatially offset inhibition despite significant differences in their dendritic morphology.

### DSGC dendritic morphology does determine the organization of spatial receptive fields

**Figure 4.**
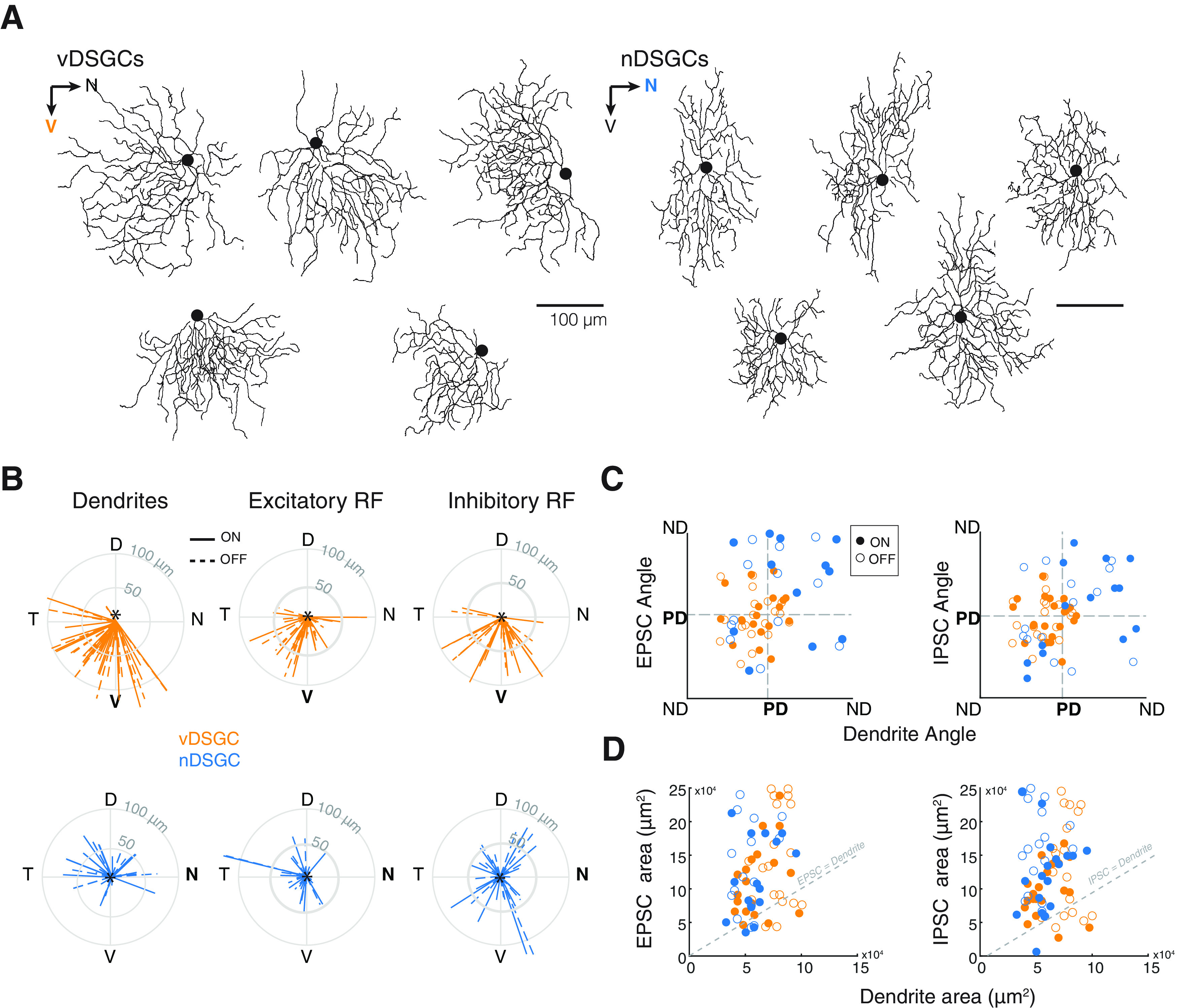
Spatial organization of receptive fields differs from dendritic morphology. ***A***, Example vDSGC (left) and nDSGC (right) dendritic skeletons. Orientation on the retina indicated by arrows, with preferred direction in bold. Scale bar: 100 μm. ***B***, Summary data represented in polar plots of the vectors from the soma to the dendrites (left), the excitatory (middle) and the inhibitory (right) receptive field COM in vDSGCs (top, orange) and nDSGCs (bottom, blue). Data for ON (solid) and OFF (dashed) plotted separately. ***C***, Summary data displaying the relationship between the orientation of dendritic morphology and the orientation of the vector from the soma to the excitatory receptive field (EPSC) COM (left) and to the inhibitory receptive field (IPSC; middle) in vDSGCs (orange) and nDSGCs (blue). Data for ON (filled circle) and OFF (open circle) plotted separately. Pearson’s correlation coefficients determined no significant correlations between dendrite angle and EPSC or IPSC locations (*p* > 0.05). ***D***, Summary data comparing the relationship between dendritic area and EPSC (left) and IPSC (right) response areas within the receptive field, and the area of the dendrites for each vDSGC (orange) and nDSGC (blue). Data for ON (filled circle) and OFF (open circle) plotted separately. Statistical significance of the EPSC/dendrite and IPSC/dendrite ratio determined with one-sided *t* test and compared with a ratio of 1 (PSC = dendrite area). All *p* values <0.001.

We next explored whether the small displacements for the EPSC and IPSC receptive field centers from the soma were correlated with variations in the spatial arrangement of the DSGC dendrites (Fig. 4*A*). To do that, we compared the distance and orientation of the COM relative to the soma of the EPSC and the IPSC peak current amplitudes of the ON and OFF responses from the soma (Fig. 2*B*) to those of the dendrites. Consistent with our previous study (El-Quessny et al., 2020), we found that the orientation of vDSGC dendrites, excitatory receptive fields, and inhibitory receptive fields were all ventrally pointing (ventral corresponds to 270°, Table 3; El-Quessny et al., 2020). In contrast, nDSGC dendrites, excitatory receptive fields, and inhibitory receptive fields did not exhibit a biased orientation toward the nasal direction (nasal corresponds to 0/360°; Fig. 4*B*,*C*; Table 3). We also found that EPSC and IPSC receptive fields were significantly larger than the dendritic fields in both vDSGCs (Fig. 4*D*, Table 3), contrary to previous studies in rabbit DSGCs (Brown et al., 2000; Yang and Masland, 1994).

**Table 3:** Summary data for Figure 4

	ON responses				OFF responses			
	vDSGCs (n = 20)		nDSGCs (n = 18)		vDSGCs		nDSGCs	
	Mean	SD	Mean	SD	Mean	SD	Mean	SD
Soma to EPSC COM vector magnitude	50.80	25.04	37.01	29.58	42.04	20.21	31.51	28.83
Soma to EPSC COM vector angle (°)	267.38	45.43	195.73	65.68	260.72	44.74	196.60	60.52
Soma to IPSC COM vector magnitude	63.00	27.20	45.05	29.69	53.89	20.79	37.50	31.94
Soma to IPSC COM vector angle (°)	264.90	41.45	295.957	74.89	259.79	42.78	279.54	75.55
EPSC area/dendrite area	1.9	0.76	2.273	1.19	1.90	0.93	2.87	1.35
IPSC area/dendrite area	1.64	0.62	2.323	1.30	1.86	0.91	3.31	1.54
	*R*	*p*	*R*	*p*	*R*	*p*	*R*	*p*
EPSC angle vs dendrite angle Pearson’s correlation	0.09	0.72	0.49	0.1	0.08	0.73	0.4	0.19
IPSC angle vs dendrite angle Pearson’s correlation	−0.08	0.74	0.78	*6e-4	0.24	0.31	0.39	0.17
EPSC area vs dendrite area Pearson’s correlation	0.35	0.14	0.35	0.24	0.13	0.59	0.21	0.50
IPSC area vs dendrite area Pearson’s correlation	0.13	0.60	0.13	0.62	0.11	0.66	−0.05	0.83

Table related to Figure 4.

*R*: correlation coefficient.

*p*: *p* value.

**Figure 5.**
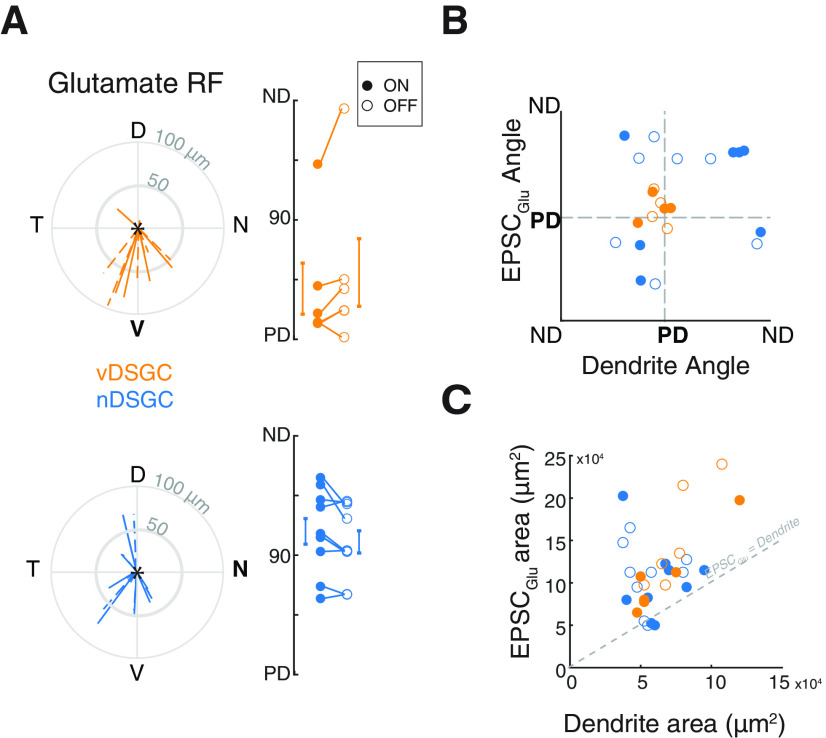
DSGC glutamatergic receptive field is more restricted to the dendritic field. ***A***, Left, Summary data represented as polar plots of the vectors from the soma to the excitatory glutamate receptive field COM in the presence of 100 μm Hexamethonium in vDSGCs (orange, top) and nDSGCs (blue, bottom) for ON (solid) and OFF (dashed) responses. Right, Summary data representing the deviation of the vector angle (right) from the vDSGC (orange, top) and nDSGC (blue, bottom) preferred direction. Data for ON (filled circle) and OFF (open circle) plotted separately. ***B***, Summary data displaying the relationship between the orientation of the vector from the soma to the glutamatergic excitatory receptive field (EPSC_Glu_) COM, relative to the orientation of the dendritic COM in vDSGCs (orange, *n* = 5 cells) and nDSGCs (blue, *n* = 9 cells). ***C***, Summary data comparing the relationship between dendritic area and the glutamatergic excitatory (EPSC_Glu_) response areas within the receptive field, and the area of the dendrites for each vDSGC (orange) and nDSGC (blue). Data for ON (filled circle) and OFF (open circle) plotted separately. Statistical significance of the EPSC_Glu_/dendrite ratio determined with one-sided *t* test and compared with a ratio of 1 (EPSC_Glu_ = dendrite area). All *p* values <0.001.

In the above experiments, EPSCs are mediated by a combination of activation of nicotinic acetylcholine receptors (nAChRs) and glutamate receptors. In a subset of experiments, where we pharmacologically blocked cholinergic excitation, we found that the orientation of the glutamate receptive field in vDSGCs was also ventrally oriented Table 4. In contrast, the orientation of the glutamate receptive field in nDSGCs was not oriented toward its preferred direction but rather, on average, was oriented toward the DSGCs’ null direction (Fig. 5*A*,*B*; Table 4). This is consistent with recent reports investigating another nDSGC subtype, where the glutamatergic receptive field was also skewed toward the DSGC’s null direction (Ding et al., 2021). Additionally, although glutamatergic receptive field were significantly larger than dendritic field size (Fig. 5*C*), they were closer in area than mixed glutamatergic-cholinergic receptive field size (compare Fig. 5*C* and *D*, left), indicating that cholinergic inputs from SACs contribute excitatory inputs outside of the DSGC dendrites. These data reveal that while asymmetric dendritic morphology of vDSGCs can predict the locations of the center of their receptive fields, dendritic field size does not dictate the size of the inhibitory or mixed excitatory receptive fields in either vDSGCs or nDSGCs.

**Table 4:** Summary data for Figure 5

	ON responses				OFF responses			
	vDSGCs (n = 5)		nDSGCs (n = 9)		vDSGCs		nDSGCs	
	Mean	SD	Mean	SD	Mean	SD	Mean	SD
Soma to EPSC_Glu_ COM vector magnitude (μm)	58.28	22.44	41.09	24.23	52.30	30.23	39.74	19.98
Soma to EPSC_Glu_ COM vector angle (°)	266.94	47.11	200.47	67.99	272.88	53.75	220.88	71.83
EPSC_Glu_ area/dendrite area	1.61	0.28	1.83	1.40	1.98	0.43	2.15	1.13
	*R*	*p*	*R*	*p*	*R*	*p*	*R*	*p*
EPSC_Glu_ area vs dendrite area Pearson’s correlation	0.96	0.003	−0.14	0.7	0.88	0.02	−0.12	0.76
IPSC area vs dendrite area Pearson’s correlation	0.13	0.60	0.13	0.62	0.11	0.66	−0.05	0.83

Table related to Figure 5.

*R*: correlation coefficient.

*p*: *p* value.

## Discussion

Dendritic morphology is thought to influence synaptic organization. Here, we show that dendritic morphology impacts the amount of tuned inhibition whereby DSGCs with asymmetric dendrites exhibit more strongly tuned inhibitory inputs than DSGCs with symmetric dendrites but both cell types exhibit comparable spatially offset inhibition. Moreover, in both cell types, we found that the receptive fields for excitation and inhibition were similarly oriented to each other and were locally correlated in strength. Finally, our results indicate that spatial receptive fields are significantly larger than dendritic fields and are not strongly dictated by the dendritic structure. Here, we discuss the implications of these findings for direction selectivity in the mouse retina.

### Asymmetric dendrites may lead to stronger tuning of inhibitory inputs

We found that vDSGCs had stronger inhibitory tuning than symmetric nDSGCs, driven primarily by a decrease in the amount of inhibition during preferred side stimulation (Fig. 1). One interpretation of these findings is that the absence of preferred side dendrites reduces the likelihood of these preferred side SAC-DSGC synapses. Serial EM reconstructions indicate that the presence of SAC-DSGC synapses is correlated with an anti-parallel organization of SAC processes and the preferred direction of the DSGC; however, this wiring rule applies across the entire dendritic tree of a symmetric DSGC (Briggman et al., 2011). Our finding that a DSGC with an asymmetric dendritic tree exhibits a relative reduction in synapses with SAC processes oriented parallel to the DSGC’s preferred direction would imply that the orientation of the dendritic branch of the DSGC itself may play a role in instructing this wiring, potentially by increasing the proportion of antiparallel compared parallel SAC-DSGC connections. Although there is no evidence for this in the adult mouse DS circuit, this scenario has not been explicitly tested. For comparison, asymmetric dendritic organization is crucial for the wiring of inputs to DS neurons in the *Drosophila*, where connectome analysis reveals dendritic asymmetry mediates the physical displacement of null and preferred side inputs (Shinomiya et al., 2019). Another example can be found in the mouse spinal cord, where the relative orientation of presynaptic and postsynaptic processes instructs circuit wiring (Balaskas et al., 2019).

Despite the difference in tuning of inhibition, vDSGCs and nDSGCs have been shown to exhibit similar spike tuning properties under our stimulus conditions (Yao et al., 2018). We think that this is because of the fact that tuning is set by the shunting inhibition generated by null direction; namely if there is sufficient inhibition, then cells will be similarly tuned (Koch et al., 1983; Taylor et al., 2000).

An alternative interpretation is that different subtypes of DSGCs receive different levels of non-DS inhibition from other sources (Pei et al., 2015; Morrie and Feller, 2018) such as VIP amacrine cells (Park et al., 2015). For example, in another population of nDSGCs, paired recordings with SACs show that asymmetric inhibition is impaired when the vesicular GABA transporter (VGAT) is knocked out from SACs, compared with wild-type animals (Pei et al., 2015). In these knock-outs, the amplitude of inhibitory input was reduced in response to null but not preferred direction stimulation, pointing to a role for non-SAC sources of inhibition during preferred direction motion.

### DSGC dendrites and the spatial organization of their receptive fields

Using receptive field mapping based on stationary stimuli, we find that there was an overall shift in the inhibitory receptive field relative to the excitatory receptive field for DSGCs with both symmetric and asymmetric dendrites. This is consistent with our previous work which has shown that vDSGCs exhibit ventrally offset inhibitory receptive fields, regardless of their altered morphology following dark rearing (El-Quessny et al., 2020).

In our present study, the spatial offset between the excitatory and inhibitory receptive fields was on average <50 μm (Fig. 2*E*), which is the resolution of our mapping. In a previous study, which uses a slightly larger stimulus to map the synaptic input receptive fields of vDSGCs, a slightly larger shift was observed in the excitatory and inhibitory receptive fields (Trenholm et al., 2013), indicating the importance of the mapping resolution in estimating spatial offset. Interestingly, the spatial offset between excitatory and inhibitory receptive fields scales with that observed in the rabbit retina, which predicted spatial offsets of 150 μm, or roughly half the dendritic tree of rabbit DSGCs (Fried et al., 2002). Given the larger dendritic field of rabbit ON-OFF DSGCs (∼600 μm in diameter; Yang and Masland, 1994; He et al. 1999; Oesch et al., 2005) compared with mouse ON-OFF DSGCs (∼200 μm in diameter; Rivlin-Etzion et al., 2011), we believe that the observed spatial offset scales with dendritic field size across both species. Additionally, the observed spatial offset is comparable but a bit smaller than predicted by the temporal offsets induced by drifting bar (Fig. 1*E*). Similarly, the displacement of the inhibitory receptive field is smaller than that predicted by the temporal offsets previously reported for symmetric nDSGCs (270 ms at 500 μm/s corresponding to 135 μm; Pei et al., 2015). This may be because of different stimulus sizes leading to differential recruitment of lateral inhibitory circuits. Another difference is that stationary stimuli may more strongly activate symmetric sources of inhibition onto DSGCs that arise from non-SACs (Morrie and Feller, 2018; Wei, 2018).

We also found that both excitatory and inhibitory receptive fields were much larger than dendritic fields (Fig. 3). Blockade of nAChR signaling reduced the size of the excitatory receptive field to that of the dendrites (Fig. 5), consistent with a larger excitatory receptive field because of cholinergic inputs from SACs (Lee et al., 2010; Sethuramanujam et al., 2016). These data are in line findings that glutamatergic receptive fields being closely aligned to the DSGC dendrite (Yang and Masland, 1994; Sethuramanujam et al., 2018; Jain et al., 2020; Rasmussen et al., 2020). Another possibility not explored here is the role of gap junctions in expanding receptive field size as recently described for F-mini ON RGCs (Cooler and Schwartz, 2021) and in vDSGCs (Trenholm et al., 2013).

It is important to note that the strength of synapses revealed by stationary receptive field mapping is different from what is activated during moving stimuli. Motion stimuli evoke directional release of GABA from SACs (Euler et al., 2002; Ding et al., 2016; Vlasits et al., 2016), and glutamate from bipolar cell terminals (Matsumoto et al., 2019). Paired recordings between SACs and DSGCs indicate that the strength of ACh synapses are symmetric, and likely mediated by diffuse release of ACh (Lee et al., 2010), while motion stimuli may lead to asymmetric release of ACh during low contrast stimuli (Poleg-Polsky and Diamond, 2016; Sethuramanujam et al., 2016). Furthermore, optogenetic stimulation of SACs expressing channelrhodopsin leads to cholinergic excitation preceding GABAergic inhibition and exhibiting faster receptor kinetics, during preferred direction motion, with all other mechanisms of synaptic inputs blocked (Hanson et al., 2019; Pottackal et al., 2020). However, receptive field mapping informs us of the overall synaptic distribution onto DSGC dendrites that could be activated by a variety of different visual stimuli. For example, a recent study has also implicated variations in the strength of excitatory receptive field, along the null-preferred axis, is critical for the ability to encode the location of moving stimuli and is revealed when the motion stimulus is interrupted by stationary occluder (Ding et al., 2021).

Here, we find that the while the glutamatergic receptive fields of nDSGCs are skewed toward the DSGC’s null direction, consistent with previous reports (Ding et al., 2021), the glutamatergic receptive fields of vDSGCs are skewed toward their preferred side. However, vDSGCs were previously reported to exhibit lag normalized synaptic responses because of gap junction coupling, enabling them to encode object location (Trenholm et al., 2013). Together, these data indicate that vDSGCs and nDSGCs may employ distinct mechanisms for encoding object location.

### Local dendritic computations support direction selectivity in DSGCs

As noted above, the extent of direction-selective tuning is set by the presence of a sufficient level of inhibition. Interestingly, there is strong evidence that the direction-selective computation is made locally on DSGC dendrites, i.e., that motion stimuli confined to small segments of the DSGC dendritic tree still elicit a directional responses (Wei, 2018). First, we found that for both vDSGCs and nDSGCs, inhibitory and excitatory receptive fields exhibiting correlated synaptic strengths (Fig. 3), indicating that regions of the receptive field with a higher number of excitatory synapses is countered by an increase in inhibitory synapses. Second, local asymmetric release of GABA are supported by the SAC plexus (Sun et al., 2013) where directional computations are localized with the SAC dendrites (Koren et al., 2017; Morrie and Feller, 2018; Poleg-Polsky et al., 2018). In fact, changes in this density of this plexus appears to be correlated with tuning whereby decreases in the coverage factor of SAC dendritic arbors (Morrie and Feller, 2018) diminishes DS tuning, while increases in the coverage factor of SAC dendritic arbors increases DS tuning (Soto et al., 2019) indicating that the density of the SAC dendritic plexus determines asymmetric inhibition of all DSGCs.

Computational modeling showed that nonlinear conductance within the dendritic tree promotes a multicompartmental model, allowing local interactions between excitation and inhibition to shape dendritic DS, while SAC ablation abolished DS (Jain et al., 2020; Sivyer and Williams, 2013). A multicompartmental model is specifically relevant for vDSGCs, whose form-function correlation enables them to nonlinearly integrate synaptic inputs along their dendrites (Trenholm et al., 2011, 2013; El-Quessny et al., 2020). This may explain how vDSGCs rely more heavily on asymmetric versus spatially offset inhibition, relative to nDSGCs which do not have a form-function relationship.

In conclusion, we show that DSGCs exhibit two parallel mechanisms for computing motion direction. The first is based on tuned inhibition, which we find is influenced by the morphology of the DSGC, and the second is based on spatially offset inhibition which is not influenced by the DSGCs’ dendritic orientation, size or asymmetry.

## Synthesis

Reviewing Editor: Siegrid Löwel, University of Goettingen Decisions are customarily a result of the Reviewing Editor and the peer reviewers coming together and discussing their recommendations until a consensus is reached. When revisions are invited, a fact-based synthesis statement explaining their decision and outlining what is needed to prepare a revision will be listed below. The following reviewer(s) agreed to reveal their identity: NONE. Note: If this manuscript was transferred from JNeurosci and a decision was made to accept the manuscript without peer review, a brief statement to this effect will instead be what is listed below.

The manuscript by El-Quessny and Feller investigates potential differences in receptive field structure and direction tuning of inhibitory input between two types of direction-selective retinal ganglion cells, which differ in their morphological structure, specifically in the asymmetry of the dendritic tree with respect to the soma position. The primary finding is that both cell types display similar spatial offsets between their excitatory and inhibitory receptive fields, but the more asymmetric type receives more strongly tuned inhibition, pointing towards a potential influence of dendritic structure on the selection of presynaptic inhibitory connections.

The manuscript poses an interesting question about the structure-function connection in certain direction-selective ganglion cells. The experimental results are solid and convincing. There are, however, two major issues with the current version of the manuscript. First, the argumentation is not always clear with respect to what is hypothesized, what is known from previous experiments, and what is deduced from the findings presented in this manuscript. This concerns, in particular, the considerations of what is expected from the asymmetric dendritic morphology around Figure 1 and the interpretation of a seemingly causal effect of morphology on inhibitory tuning. Second, the description of the Methods is somewhat cursory. Details are given below.

1) Hypothesized effects of asymmetric geometry. The manuscript is largely motivated by the hypotheses that are drawn from Figure 1. In particular, it is hypothesized that an asymmetric dendritic tree leads to a different selection of presynaptic inhibitory input, and this hypothesis seems to be derived from or explained by geometrical considerations of dendritic overlap with starburst amacrine cells (SACs). However, it is not clear why the dendritic asymmetry and the selection of inhibitory input should be connected at all. If one takes out the soma position of the ganglion cell and only considers the two roughly circular dendritic fields of the symmetric and asymmetric ganglion cells, the two scenarios would be identical. This point had already been raised by Reviewer 2 in the previous round (Reviewer’s major comment 1), and it seems to have been insufficiently addressed. Maybe the authors have something else in mind, like an influence of the orientation of individual dendritic arbors or a deviation from circular dendritic fields, but then this should be laid out, and it should be more clearly differentiated what is hypothesized and what is deduced. Perhaps the simplified layout of Figure 1 with only two SACs at nearly fixed distances from the ganglion cell soma is a bit too suggestive. Null-side SACs, for example, likely also make synaptic contacts on the opposite side of the ganglion cell dendritic tree (e.g. when the soma is closer to the ganglion cell soma). The differentiation of hypotheses and deductions is relevant for motivating the investigations at the outset, but also becomes important for the Discussion because a similar logic seems to be invoked when interpreting the difference in inhibitory tuning (see point below).

2) Interpretation of difference in inhibitory tuning. The wording in the text suggests a causal link between the different dendritic geometries and the difference in inhibitory tuning between the two investigated ganglion cell types. (E.g. Abstract: “morphology influences the amount of tuned inhibition attained through asymmetric wiring", p. 9: “we show that dendritic morphology impacts the amount of tuned inhibition", p. 13: “tuned inhibition, which we find is influenced by the morphology of the DSGC”.) Yet, the data only show a correlation in the sense that the more asymmetric type has stronger inhibitory tuning. This seems to be connected to the seemingly simple mechanistic explanation that “the absence of preferred side dendrites reduces the likelihood of the preferred side SAC-DSGC synapses” (p. 10), an argument that goes back to the unclear argumentation around Figure 1 (see point above) and doesn’t seem to hold up based on geometrical reasoning alone. Again, this is a point that Reviewer 2 already made in the previous round (Reviewer’s major comment 2). The Reviewer also suggested to consider (at least) one alternative mechanism, namely different levels of untuned inhibition. The authors picked this up, but simply state (without explanation) that they consider it unlikely, which is puzzling.

3) There are a few other sentences in the Discussion where the argumentation is unclear (or perhaps just unfortunately phrased), which may also be related to the assumed differences in presynaptic inhibitory connections, following the dendritic asymmetry. Although I read the corresponding sections several times, the logic still eluded me, so I’d like to suggest clarification and perhaps simpler wording. E.g.: “the high coverage factor of SAC processes enables processes from nearby SACs to asymmetrically wire onto DSGCs” (p. 10), “the dense SAC dendritic plexus combined with their axon-less morphology and non-spiking physiology necessitates alternative methods for generating spatially offset inhibition” (p. 11), “Since motion stimuli more efficiently activate presynaptic mechanisms for DS, evoking directional release of GABA, and glutamate onto DSGC dendrites, our study demonstrates that temporal offsets in excitation and inhibition do not necessarily reflect spatial offsets in synaptic input distribution” (p. 12), “However, our receptive field mapping experiments have revealed a similar size of mixed excitatory (Ach+Glutamate) and inhibitory receptive fields indicating that although the anatomical map of excitation and inhibition may not reflect the functional asymmetries in synaptic wiring, and presynaptic SAC computations elicited by a moving stimulus, they still inform us of the overall synaptic distribution of DSGC dendrites” (p. 12).

4) In response to another comment from Reviewer 2, the manuscript now explains that the spiking output of the two ganglion cell types shows similar direction tuning. This raises the questions why the observed differences in inhibitory tuning seem to have no effect on the spiking responses and what the relevance of the inhibitory tuning difference then is. Is it canceled by some other difference between the cell types? Does it become important for other types of motion stimuli? Maybe the authors would like to speculate about the potential role of the different inhibitory tunings.

5) Description of visual stimuli: The parameters of the applied visual stimuli should be more clearly specified. E.g. were the drifting bars of positive as well as negative contrast? On a gray background? What contrast value? Moving along the long or short axis of the bar? Temporal separation between individual bars? Similarly for the receptive field mapping: Positive as well as negative contrast? Individual presentations for how long? Separated by how much time? Randomization?

6) Description of data analysis: How were the centers of mass of the receptive fields computed? How were the receptive field sizes computed? How were the measured receptive fields aligned with the imaged ganglion cell to compute the offset between soma and receptive field center? How were On and Off responses combined into a single preferred direction?

7) The text references several tables, but these seem not to be included in the submission.

Minor comments:

p. 3 “asymmetric dendrites that are oriented toward their preferred direction, a configuration which contributes to their direction selectivity”: In which way does this contribute? Didn’t the authors’ previous paper with dark-reared animals show that direction selectivity is retained even if the asymmetry is gone?

p. 5 “GW seal”: Should this be “GOhm seal”?

p. 5: It seems surprising that Alexa 594 is activated well with 800 nm. Is this correct? Isn’t the excitation peak near 600 nm?

## Author Response

Synthesis Statement for Author (Required):

The manuscript by El-Quessny and Feller investigates potential differences in receptive field

structure and direction tuning of inhibitory input between two types of direction-selective retinal

ganglion cells, which differ in their morphological structure, specifically in the asymmetry of the

dendritic tree with respect to the soma position. The primary finding is that both cell types

display similar spatial offsets between their excitatory and inhibitory receptive fields, but the

more asymmetric type receives more strongly tuned inhibition, pointing towards a potential

influence of dendritic structure on the selection of presynaptic inhibitory connections.

The manuscript poses an interesting question about the structure-function connection in certain

direction-selective ganglion cells. The experimental results are solid and convincing. There are,

however, two major issues with the current version of the manuscript. First, the argumentation is

not always clear with respect to what is hypothesized, what is known from previous

experiments, and what is deduced from the findings presented in this manuscript. This

concerns, in particular, the considerations of what is expected from the asymmetric dendritic

morphology around Figure 1 and the interpretation of a seemingly causal effect of morphology

on inhibitory tuning. Second, the description of the Methods is somewhat cursory. Details are

given below.

We thank reviewers and editors for their tremendous effort on the manuscript. We have

eliminated Figure 1 (see comments below) and added the details to the Methods. Our

responses to these extensive comments may appear brief but we have implemented all of them

in the revised manuscript.

1) Hypothesized effects of asymmetric geometry. The manuscript is largely motivated by the

hypotheses that are drawn from Figure 1. In particular, it is hypothesized that an asymmetric

dendritic tree leads to a different selection of presynaptic inhibitory input, and this hypothesis

seems to be derived from or explained by geometrical considerations of dendritic overlap with

starburst amacrine cells (SACs). However, it is not clear why the dendritic asymmetry and the

selection of inhibitory input should be connected at all. If one takes out the soma position of the

ganglion cell and only considers the two roughly circular dendritic fields of the symmetric and

asymmetric ganglion cells, the two scenarios would be identical. This point had already been

raised by Reviewer 2 in the previous round (Reviewer’s major comment 1), and it seems to have

been insufficiently addressed. Maybe the authors have something else in mind, like an influence

of the orientation of individual dendritic arbors or a deviation from circular dendritic fields, but

then this should be laid out, and it should be more clearly differentiated what is hypothesized

and what is deduced. Perhaps the simplified layout of Figure 1 with only two SACs at nearly

fixed distances from the ganglion cell soma is a bit too suggestive. Null-side SACs, for example,

likely also make synaptic contacts on the opposite side of the ganglion cell dendritic tree (e.g.

when the soma is closer to the ganglion cell soma). The differentiation of hypotheses and

deductions is relevant for motivating the investigations at the outset, but also becomes

important for the Discussion because a similar logic seems to be invoked when interpreting the

difference in inhibitory tuning (see point below).

We have now gone through several reviewers (and we have had subsequent extensive

conversations with a few DS experts) and we are convinced that what we had proposed is a

very unlikely hypothesis and therefore should not be the primary motivation of the paper.

We have eliminated Figure 1 and motivated our study more generally. We have also revised the

discussion significantly to simplify and hopefully clarify our hypotheses. Please see response to

next comment for more details.

2) Interpretation of difference in inhibitory tuning. The wording in the text suggests a causal link

between the different dendritic geometries and the difference in inhibitory tuning between the

two investigated ganglion cell types. (E.g. Abstract: “morphology influences the amount of tuned

inhibition attained through asymmetric wiring", p. 9: “we show that dendritic morphology impacts

the amount of tuned inhibition", p. 13: “tuned inhibition, which we find is influenced by the

morphology of the DSGC”.) Yet, the data only show a correlation in the sense that the more

asymmetric type has stronger inhibitory tuning. This seems to be connected to the seemingly

simple mechanistic explanation that “the absence of preferred side dendrites reduces the

likelihood of the preferred side SAC-DSGC synapses” (p. 10), an argument that goes back to

the unclear argumentation around Figure 1 (see point above) and doesn’t seem to hold up

based on geometrical reasoning alone. Again, this is a point that Reviewer 2 already made in

the previous round (Reviewer’s major comment 2). The Reviewer also suggested to consider (at

least) one alternative mechanism, namely different levels of untuned inhibition. The authors

picked this up, but simply state (without explanation) that they consider it unlikely, which is

puzzling.

I have exerpted part of the revised discussion (page 10) here. Note we have added the

motivation of this interpretation from studies on other species/region of nervous system (in bold

below). For what it is worth, these were indeed the motivations of this study - to see if

orientation of dendrites instructs wiring. In the revision, we try to make clear that this is

speculation on our part. Note, we have also eliminated the sentence stating that different levels

of untuned inhibition are “unlikely”.

We found that vDSGCs had stronger inhibitory tuning than symmetric nDSGCs, driven

primarily by a decrease in the amount of inhibition during preferred side stimulation

(Figure 1). One interpretation of these findings is that the absence of preferred side

dendrites reduces the likelihood of these preferred side SAC-DSGC synapses. Serial EM

reconstructions indicate that the presence of SAC-DSGC synapses is correlated with an

anti-parallel organization of SAC processes and the preferred direction of the DSGC,

however, this wiring rule applies across the entire dendritic tree of a symmetric DSGC

(Briggman et al., 2011). Our finding that a DSGC with an asymmetric dendritic tree

exhibits a relative reduction in synapses with SAC processes oriented parallel to the

DSGC’s preferred direction would imply that the orientation of the dendritic branch of

the DSGC itself may play a role in instructing this wiring, potentially by increasing the

proportion of antiparallel compared parallel SAC-DSGC connections. Though there is no

evidence for this in the adult mouse DS circuit, this scenario has not been explicitly

tested. For comparison, asymmetric dendritic organization is crucial for the wiring of

inputs to DS neurons in the drosophila, where connectome analysis reveals dendritic

asymmetry mediates the physical displacement of null and preferred side inputs

(Shinomiya et al., 2019). Another example can be found in the mouse spinal cord,

where the relative orientation of pre-and postsynaptic processes instructs circuit

wiring (Balaskas et al., 2019).

An alternative interpretation is that different subtypes of DSGCs receive different levels

of non-DS inhibition from other sources....

3) There are a few other sentences in the Discussion where the argumentation is unclear (or

perhaps just unfortunately phrased), which may also be related to the assumed differences in

presynaptic inhibitory connections, following the dendritic asymmetry. Although I read the

corresponding sections several times, the logic still eluded me, so I’d like to suggest clarification

and perhaps simpler wording. E.g.: “the high coverage factor of SAC processes enables

processes from nearby SACs to asymmetrically wire onto DSGCs” (p. 10), “the dense SAC

dendritic plexus combined with their axon-less morphology and non-spiking physiology

necessitates alternative methods for generating spatially offset inhibition” (p. 11), “Since motion

stimuli more efficiently activate presynaptic mechanisms for DS, evoking directional release of

GABA, and glutamate onto DSGC dendrites, our study demonstrates that temporal offsets in

excitation and inhibition do not necessarily reflect spatial offsets in synaptic input distribution” (p.

12), “However, our receptive field mapping experiments have revealed a similar size of mixed

excitatory (Ach+Glutamate) and inhibitory receptive fields indicating that although the

anatomical map of excitation and inhibition may not reflect the functional asymmetries in

synaptic wiring, and presynaptic SAC computations elicited by a moving stimulus, they still

inform us of the overall synaptic distribution of DSGC dendrites” (p. 12).

We have greatly simplified the discussion and these sentences have either been revised or

eliminated.

4) In response to another comment from Reviewer 2, the manuscript now explains that the

spiking output of the two ganglion cell types shows similar direction tuning. This raises the

questions why the observed differences in inhibitory tuning seem to have no effect on the

spiking responses and what the relevance of the inhibitory tuning difference then is. Is it

canceled by some other difference between the cell types? Does it become important for other

types of motion stimuli? Maybe the authors would like to speculate about the potential role of

the different inhibitory tunings.

This is the basis of the revised discussion (page 10).

Despite the different in tuning of inhibition, vDSGCs and nDSGCs have been shown to

exhibit similar spike tuning properties under our stimulus conditions (Yao et al., 2018).

We think that this is due to the fact that tuning is set by the inhibition generated by null

direction; namely if there is sufficient inhibition, then cells will be similarly tuned (Koch et

al., 1983; Taylor et al., 2000).

In addition, we have modified the section entitled - “ Local dendritic computations support

direction selectivity in DSGCs” to speculate how this is implemented locally of DSGC dendrites.

Though we haven’t addressed all of the interesting questions raised by the reviewer, we think it

provides sufficient perspective.

5) Description of visual stimuli: The parameters of the applied visual stimuli should be more

clearly specified. E.g. were the drifting bars of positive as well as negative contrast? On a gray

background? What contrast value? Moving along the long or short axis of the bar? Temporal

separation between individual bars? Similarly, for the receptive field mapping: Positive as well

as negative contrast? Individual presentations for how long? Separated by how much time?

Randomization?

We have added these details in the revised (and expanded) methods section.

6) Description of data analysis: How were the centers of mass of the receptive fields computed?

How were the receptive field sizes computed? How were the measured receptive fields aligned

with the imaged ganglion cell to compute the offset between soma and receptive field center?

How were On and Off responses combined into a single preferred direction?

We have added these details in the revised methods section.

7) The text references several tables, but these seem not to be included in the submission.

We apologize for the error. Tables are now included in the main text.

Minor comments:

p. 3 “asymmetric dendrites that are oriented toward their preferred direction, a configuration

which contributes to their direction selectivity”: In which way does this contribute? Didn’t the

authors’ previous paper with dark-reared animals show that direction selectivity is retained even

if the asymmetry is gone?

We modified the text to state that the dendritic contribution to direction selectivity is unmasked in

the absence of inhibitory inputs (pg. 3, para. 2).

p. 5 “GW seal”: Should this be “GOhm seal”?

We have made this change.

p. 5: It seems surprising that Alexa 594 is activated well with 800 nm. Is this correct? Isn’t the

excitation peak near 600 nm?

Yes, the single-photon excitation peak of Alexa 594 is around 600 nm, but the two-photon

excitation peak of Alexa 594 is around 810 nm, according to measurements made by

ThermoFisher Scientific (the suppliers of the dye). We use two-photon excitation for all our

imaging to avoid bleaching the retinal photoreceptors.
